# Fibroblast Growth Factor 2 Conjugated with Monomethyl Auristatin E Inhibits Tumor Growth in a Mouse Model

**DOI:** 10.1021/acs.biomac.1c00662

**Published:** 2021-09-20

**Authors:** Mateusz A. Krzyscik, Malgorzata Zakrzewska, Vigdis Sørensen, Geir Frode Øy, Skjalg Brunheim, Ellen M. Haugsten, Gunhild M. Mælandsmo, Antoni Wiedlocha, Jacek Otlewski

**Affiliations:** † Department of Protein Engineering, Faculty of Biotechnology, University of Wroclaw, Joliot-Curie 14a, Wroclaw 50-383, Poland; ‡ Advanced Light Microscopy Core Facility, Dept. Core Facilities, Institute for Cancer Research, The Norwegian Radium Hospital, Oslo University Hospital, Montebello, Oslo 0379, Norway; § Centre for Cancer Cell Reprogramming, Institute of Clinical Medicine, Faculty of Medicine, University of Oslo, Montebello, Oslo 0379, Norway; ∥ Department of Tumor Biology, Institute for Cancer Research, The Norwegian Radium Hospital, Oslo University Hospital, Montebello, Oslo 0379, Norway; ⊥ Department of Molecular Cell Biology, Institute for Cancer Research, The Norwegian Radium Hospital, Oslo University Hospital, Montebello, Oslo 0379, Norway; # University in Tromso - Arctic University of Norway, Tromso 9019, Norway; ∇ Military Institute of Hygiene and Epidemiology, Warsaw 01-163, Poland

## Abstract

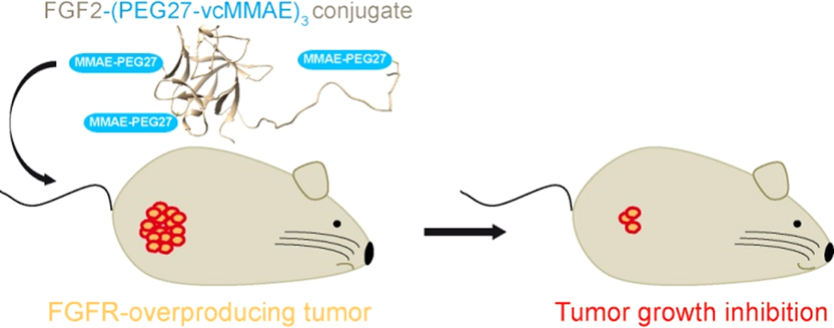

Worldwide, cancer is the second leading cause of death. Regardless of the continuous progress in medicine, we still do not have a fully effective anti-cancer therapy. Therefore, the search for new targeted anti-cancer drugs is still an unmet need. Here, we present novel protein–drug conjugates that inhibit tumor growth in a mouse model of human breast cancer. We developed conjugates based on fibroblast growth factor (FGF2) with improved biophysical and biological properties for the efficient killing of cancer cells overproducing fibroblast growth factor receptor 1 (FGFR1). We used hydrophilic and biocompatible PEG4 or PEG27 molecules as a spacer between FGF2 and the toxic agent monomethyl auristatin E. All conjugates exhibited a cytotoxic effect on FGFR1-positive cancer cell lines. The conjugate with the highest hydrodynamic size (42 kDa) and cytotoxicity was found to efficiently inhibit tumor growth in a mouse model of human breast cancer.

## Introduction

Cancer treatment is one of the major areas of research in current medicine. Nevertheless, only a limited number of chemotherapeutics are available for treatment, and even fewer show significant clinical benefits.^1−4^ Therefore, the search for new anticancer therapies is an ever-present need, and targeted therapy is a promising approach that can meet these expectations. One of the main strategies in targeted therapy is the use of antibody–drug conjugates (ADCs). In ADC, a monoclonal antibody (mAb), as a targeting molecule, is conjugated to a highly potent cytotoxic drug that kills cancer cells. Recently, only a few ADCs, including ado-trastuzumab emtansine (T-DM1, Kadcyla), brentuximab vedotin (Adcetris), gemtuzumab ozogamicin (Mylotarg), inotuzumab ozogamicin (Besponsa), and polatuzumab vedotin-piiq (Polivy), have been approved for treatment by the FDA.^5−8^ This shows that despite the high potential of this strategy, further studies are needed. One possible way forward for such an approach is to use proteins other than monoclonal antibodies as targeting molecules.

Previously, we have shown that fibroblast growth factors 1 and 2 (FGF1 and FGF2) are suitable targeting molecules for killing cancer cells overproducing fibroblast growth factor receptor 1 (FGFR1).^9−17^ FGFR1 is a transmembrane protein that plays a substantial role in regulating cell proliferation, survival, differentiation, migration, and angiogenesis.^18−23^ Analyses have shown that 7.1% of all cancer types are associated with aberration of the FGF-FGFR pathway, with FGFR1 being the most commonly affected.^24,25^ Upregulation of FGFR1 occurs in many types of tumors, including bladder, breast, lungs, multiple myeloma, pancreatic, prostate, and various sarcomas.^18,21,25−32^ Furthermore, overexpression of FGFR1 is correlated with poor prognosis.^33,34^ Thus, the development of therapy based on FGF2 conjugates targeting FGFR1 is most warranted. FGF2, however, is a relatively small protein (17.2 kDa), which does not have adequate pharmacokinetic properties. Therefore, modifications that increase the effective size and in vivo stability, thereby prolonging the systemic half-life of the therapeutic macromolecule, are required. The widely applied approaches use PEGylation, PASylation, and conjugation to other proteins, such as albumin.^35−37^ PEGylation is a particularly preferred and common method as it significantly improves solubility and hydrodynamic radius and reduces immunogenicity, sensibility to proteolysis, and renal elimination.^38−40^ It should be noted, however, that in many cases PEGylation reduces the affinity to the molecular target.^41,42^

Here, we developed FGF2-based conjugates with improved biophysical and biological properties by applying a site- and stoichiometric-controlled conjugation of PEGylated monomethyl auristatin E (MMAE) to FGF2. PEGylation does not impair the biological activities of the FGF2 conjugate, such as affinity to the molecular target (FGFR1), and the ability to activate FGF-induced signaling and to be internalized by the receptor-dependent pathway. Taken together, the presented conjugates have increased hydrophilicity and a larger hydrodynamic size, compared to non-PEGylated constructs. Of outmost importance, they exhibit high toxicity against FGFR1-overproducing cancer cells in vitro and show efficient tumor growth retardation in an FGFR-positive human breast cancer xenograft model in mice.

## Experimental Section

### Materials

#### Reagents and Antibodies

All chemical reagents were obtained from commercial suppliers and were used without further purification. l-Cysteine was purchased from BioShop (Burlington, ON). mc-vc-PAB-MMAE (HY-15575) and monomethyl auristatin E (HY-15162) were obtained from MedChemExpress (Monmouth Junction, NJ). mal-dPEG(4)-NHS (PEG1575), mal-dPEG(24)-NHS (PEG1565), *N,N*-diisopropylethylamine (DIPEA), and trifluoroacetic acid (TFA) were purchased from Iris Biotech GmbH (Marktredwitz, Germany). High-performance liquid chromatography (HPLC)-pure acetonitrile was purchased from Avantor (Gliwice, Poland), and *N,N*-dimethylacetamide (DMAc) was obtained from Sigma-Aldrich (Merck KGaA, Darmstadt, Germany). The chromatographic columns HiTrap Desalting with Sephadex G-25 resin, Superdex 75 10/300 GL, HiTrap CM Sepharose FF, and HiTrap Heparin HP were obtained from GE Healthcare (UK). Zeba spin desalting columns were obtained from Thermo Fisher Scientific (Waltham, MA), and Aeris PEPTIDE 3.6 μm XB-C18 250 × 4.6 mm and Synergi 4 μm Fusion-RP 80 Å 250 × 10 mm LC columns were obtained from Phenomenex Inc. (Torrance, CA).

The following antibodies were used: mouse anti-phospho-FGF Receptor (Tyr653/654) (#3476), rabbit anti-FGF Receptor 1 (#9740), rabbit anti-p44/42 MAPK (#9102), and mouse anti-phospho-p44/42 MAPK (Thr202/Tyr204) (#9106) from Cell Signaling Technology (Danvers, MA), mouse anti-γ-tubulin (T6557) from Sigma-Aldrich (St. Louis, MO), goat anti-FGF2 antibody (sc1390) from Santa Cruz Biotechnology (Dallas, TX), rabbit anti-FGFR1(ab76464) from Abcam (UK), mouse anti-EEA1 (early endosomal antigen protein-1, #610456) from BD Transduction Laboratories (San Jose, CA), and goat anti-mouse (115-035-003), anti-rabbit (111-035-144) conjugated to HRP, and donkey anti-goat, anti-rabbit, and anti-mouse coupled to the fluorophores Alexa Fluor-488, Alexa Fluor-568, or Alexa Fluor-647 were obtained from Jackson ImmunoResearch Europe Ltd. (UK). Annexin V-FITC apoptosis detection kit was obtained from Thermo Fisher Scientific (Waltham, MA). All other reagents were obtained from Sigma-Aldrich (Saint Louis, MO), Thermo Fisher Scientific (Waltham, MA), or BioShop Canada Inc. (Burlington, ON).

#### Cells

DMS114 cells (human small cell lung cancer, ATCC CRL-2066) obtained from American Type Culture Collection (Manassas, VA) were cultured in the Waymouth’s MB 752/1 medium from Gibco (Waltham, MA). MCF7 cells (human adenocarcinoma, ATCC HTB-22) obtained from American Type Culture Collection were cultured in DMEM high glucose with stable glutamine and sodium pyruvate from Biowest (France). The MCF7 cells stably expressing FGFR1 (MCF7-R1) were generated by transfection of pcDNA3-FGFR1 using DOTAP (Roche Diagnostics). Clones were selected with 1 mg/mL G-418 and chosen based on their receptor expression level analyzed by immunofluorescence and immunoblotting. MCF7-R1 cells were cultured in DMEM high glucose (Biowest) with 50 μg/mL gentamicin sulfate (Thermo Fisher Scientific), stable glutamine, and sodium pyruvate (Biowest).

### Methods

#### Synthesis of PEGylated MMAE Moieties

#### Step 1—Synthesis and Purification of l-Cys-MMAE

l-Cysteine (184 mg, 1.52 mmol, 20 equiv), maleimidocaproyl-valine-citrulline-*p*-aminobenzoyloxycarbonyl-monomethyl auristatin E (100 mg, 0.08 mmol), and DIPEA (26.5 μL, 0.16 mmol, 2 equiv) were added to 1 mL of DMAc. The reaction was conducted at 30 °C for 12 h. Next, the solvent was removed under vacuum. The crude product was dissolved in 500 μL of 30% acetonitrile/water with 0.1% TFA, and then the final product was separated from excess of Cys by reversed-phase (RP)-HPLC. Next, the solvent was removed by lyophilization.

#### Step 2a—Synthesis and Purification of Maleimide-PEG4-MMAE

To a solution of l-Cys-MMAE (50 mg, 0.035 mmol) in 500 μL of DMAc was added mal-dPEG(4)-NHS (89.9 mg, 0.175 mmol, 5 equiv) and DIPEA (12.4 μL, 0.075 mmol, 2 equiv). The reaction mixture was incubated at 30 °C for 12 h. Next, the solvent was removed under vacuum. The crude product was dissolved in 500 μL of 30% acetonitrile/water with 0.1% TFA, and then the final product was separated from excess of Cys by RP-HPLC followed by solvent removal by lyophilization.

#### Step 2b—Synthesis and Purification of Maleimide-PEG27-MMAE

To a solution of l-Cys-MMAE (50 mg, 0.035 mmol) in 500 μL of DMAc was added mal-dPEG(27)-NHS (274.8 mg, 0.175 mmol, 5 equiv) and DIPEA (12.4 μL, 0.075 mmol, 2 equiv). The reaction mixture was incubated at 30 °C for 12 h. Next, the solvent was removed under vacuum. The crude product was dissolved in 500 μL of 30% acetonitrile/water with 0.1% TFA, and then the final product was separated from excess of Cys by RP-HPLC. Next, the solvent was removed by lyophilization.

### Protein Production and Purification

The pET-3c plasmid encoding fibroblast growth factor 2 with N-terminal KCKSGG and the *E. coli* Rosetta 2(DE3)pLysS expression strain from Novagen-EMD Biosciences (Madison, WI) were used to express the recombinant protein.^12^ Protein production was carried out in the BIOSTAT C fermentor system (B. Braun Biotech International, Germany). Bacteria were grown to OD_600_ = 8 in a TB medium with 100 μg/mL ampicillin at 37 °C, pO_2_ = 35–50%, and a stirring speed of 250 rpm. Then, the temperature was decreased to 20 °C, and the protein production was induced by adding IPTG to a final concentration of 0.5 mM and continued for 12 h. After this time, bacteria were harvested by centrifugation at 6500 *g*, resuspended in a lysis buffer (50 mM monosodium phosphate, 0.15 M NaCl, 1 mM dithiothreitol (DTT), 1 mM ethylenediaminetetraacetic acid (EDTA), 0.1% Triton X-100, 1 mM PMSF, pH 7.2) supplemented with 500 U/L of Pierce Universal Nuclease (Thermo Fisher Scientific), and homogenized using a French press. The cell debris was separated by ultracentrifugation at 50,000 *g* at 4 °C for 1 h. The clarified cell lysate was diluted in 50 mM monosodium phosphate, 0.7 M NaCl, 10 mM (NH_4_)_2_SO_4_, 1 mM DTT, 1 mM EDTA, pH 7.2 and loaded on a HiTrap Heparin HP column. The column was washed with a washing buffer (50 mM monosodium phosphate, 1.0 M NaCl, 10 mM (NH_4_)_2_SO_4_, 1 mM DTT, 1 mM EDTA, pH 7.2), and proteins were eluted with a linear 1.0–2.0 M gradient of NaCl in the same buffer.

### Conjugation of MMAE and PEGylated MMAEs to FGF2

MMAE, maleimide-PEG4-MMAE, and maleimide-PEG27-MMAE were dissolved in DMAc at a concentration of 50 mg/mL. Attachment of a cytotoxic payload containing a maleimide moiety to the sulfhydryl group of the protein was performed in the reaction buffer (25 mM monosodium phosphate, 10 mM Na_2_SO_4_, 10 mM methionine, 1 mM EDTA, pH 7.0) at a protein concentration of 0.5 mg/mL and fivefold molar excess of payload per sulfhydryl group. The reaction was performed at 20 °C for 1 h. The conjugates were then purified using the HiTrap CM Sepharose column. The reaction mixtures were loaded onto the column, the unreacted payload was washed with 25 mM monosodium phosphate and 10 mM Na_2_SO_4_, and finally the conjugates were eluted with the same buffer containing 0.5 M NaCl.

### RP-HPLC

To evaluate the conjugation yield and homogeneity of products, the RP-HPLC analysis was performed using the 1260 Infinity Nanoflow LC system (Agilent Technologies, CA) and a C18 column (Aeris PEPTIDE 3.6 μm XB-C18 250 × 4.6 mm, Phenomenex Inc., Torrance, CA) with 35–45% gradient of water–acetonitrile supplemented with 0.1% TFA. Absorption measurements at 280 nm were used to detect the conjugates.

### Mass Spectrometry (MS)

The molecular masses of protein and conjugates were determined by matrix-assisted laser desorption/ionization–time-of-flight–mass spectrometry (MALDI-TOF-MS, AB 4800+, Applied Biosystems, Waltham, MA) using α-cyano-4-hydroxycinnamic acid as the matrix.

### Spectrofluorimetry

To validate the folded state of the protein and conjugates, spectrofluorimetric measurements were performed. Fluorescence spectra were acquired using an FP-8500 spectrofluorimeter (Jasco, Japan) with excitation at 280 nm and emission in the range of 300–450 nm at a sample concentration of 4 × 10^–6^ M in a size-exclusion chromatography (SEC) buffer at 20 °C.

### SEC

SEC was performed to estimate the hydrodynamic radius-based molecular mass of FGF2 and conjugates. The analysis was carried out at 20 °C using an ÄKTA Explorer FPLC system (GE Healthcare, UK) with a Superdex 75 HR 10/30 column. Samples at a concentration of 2.5 mg/mL were loaded onto the column using a full 50 μL loop injection. The mobile phase (25 mM monosodium phosphate, pH = 7.4; 10 mM Na_2_SO_4_) was pumped at a flow rate of 1 mL/min, and the absorption at 280 nm was recorded. Molecular weight standards containing BPTI, cytochrome C, carbonic anhydrases, human serum albumin, α-lactoglobulin, chymotrypsinogen A, ovalbumin, and albumin were used to generate a standard curve from which the effective size of the PEGylated conjugates was estimated.

### Dynamic Light Scattering (DLS)

DLS experiments were performed to evaluate the behavior of conjugates in solution. Measurements were performed using a DynaPro NanoStar instrument (Wyatt Technology, CA) equipped with a 658 nm (red) laser. A disposable microcuvette (Wyatt Technology) was used. Each measurement was performed at 20 °C in the buffer described in the SEC analysis and it involved 12, 5 s runs. DLS data were collected and analyzed using DYNAMICS V7 software (Wyatt Technology). All DLS-based hydrodynamic diameters and molecular masses were determined by cumulant analysis using the Rayleigh sphere model.

### Bio-Layer Interferometry (BLI)

To confirm the interaction of FGF2 after conjugation with extracellular domains of FGFR1, kinetic rate constants were measured. ForteBio Octet K2 (Pall ForteBio, CA) and high-precision Streptavidin biosensors (SAX) (Pall ForteBio) were used. Studies of binding between biotinylated extracellular domains of FGFR1c fused to Fc fragments and FGF2 or conjugates were performed in a similar manner to that reported previously.^12^ Association at different concentrations (40, 60, and 80 nM) was carried out for 300 s, and the dissociation was monitored for 300 s. Kinetic parameters were calculated using a 1:1 model with Octet Data Analysis software 9.0.

### Activation of FGF2 Signaling Pathways

We analyzed FGF2-induced signaling pathways to confirm the ability of FGF2 within conjugates to perform its primary biological function.^12^ Briefly, serum-starved NIH 3T3 cells were incubated for 15 min with 0.1, 1, 5, 10, or 15 ng/mL FGF2 WT, FGF2, or their conjugates in the presence of heparin (10 U/mL). Then, the whole cell lysate was separated by sodium dodecyl sulfate–polyacrylamide gel electrophoresis (SDS-PAGE), transferred onto a PVDF membrane, and analyzed using the following antibodies: anti-phospho-FGFR, anti-FGFR1, anti-phospho-p44/42 MAPK, anti-p44/42 MAPK, and anti-γ-tubulin. All primary antibodies were used at a 1:1000 dilution. Specific protein bands were visualized with HRP-conjugated secondary antibodies and a chemiluminescent substrate using a ChemiDoc station (Bio-Rad, Hercules, CA).

### Wide Field Immunofluorescence Microscopy

To test whether the FGF2 conjugates can be taken up by receptor-mediated endocytosis in FGFR-expressing cells, we used MCF7 cells stably expressing FGFR1 (MCF7-R1). The cells, grown on glass coverslips, were incubated with 300 ng/mL unconjugated FGF2 or FGF2-(PEG27-MMAE)_3_ conjugate in the presence of 50 U/mL heparin in an HEPES medium at 4 °C for 1 h to allow binding of the FGF2 ligands to cell surface receptors. In addition, as an additive control, MCF7-R1 cells were preincubated on ice with 10-fold excess (3 μg/mL) of FGF1 before the addition of the FGF2-(PEG27-MMAE)_3_ conjugate. The cells were then incubated at 37 °C for 40 min to allow endocytosis and then fixed with 4% formaldehyde in a phosphate-buffered saline (PBS) buffer. The fixed cells were treated with 0.05% Saponin for permeabilization and then stained with the following antibodies: goat anti-FGF2, rabbit anti-FGFR1, and mouse anti-EEA1 and then with the secondary antibodies donkey anti-goat, anti-rabbit, and anti-mouse coupled to fluorophores Alexa Fluor-488, Alexa Fluor-568, or Alexa Fluor-647, respectively, and Hoechst33342 for DNA staining. The stained cells were mounted in a ProLong gold antifade mountant (Thermo Fisher) and imaged using a DeltaVision OMX V4 microscope (GE Health Care) equipped with an Olympus ×60 NA 1.42 plan apochromat objective, an InsightSSI wide field illumination module, and three cooled sCMOS cameras. Four-channel (color) images, including z-stacks covering the entire cell of interest, were recorded. Raw data images were deconvolved and aligned using Softworx software (Applied Precision Inc.). For illustrations, a single z-section (optical section) was selected, and images were prepared using Fiji software.^43^

### Analysis of the Expression Level of FGFR1 and the Internalization Efficiency of FGF2 and Its Conjugates

Analysis of FGFR1 levels in MCF7-R1, MCF7, and DMS114 cell lines was performed by Western blotting as previously reported.^12^ Quantification of internalization of FGF2 and its conjugates into MCF7-R1 cells was carried out using flow cytometry and fluorescence microscopy.^16^

### Analysis of the Mechanism of Cell Death

Analysis of the mechanism of cell death was carried out as previously described.^44^ Briefly, 100,000 MCF7-R1 cells were seeded into each well of a 12-well culture plate, allowed to adhere overnight, and treated with 10 nM MMAE or conjugates in the presence of 10 U/mL heparin for 72 h. Then the cells were harvested with a TrypLE Express solution (Thermo Fisher Scientific), stained by Annexin V-FITC and propidium iodide (according to the manufacturer’s protocol), and analyzed by flow cytometry using a NovoCyte 2060R flow cytometer (ACEA Biosciences, San Diego, CA).

### Cell Viability Assay

Conjugate toxicity was quantitatively measured using the resazurin-based alamarBlue reagent (Thermo Fisher Scientific, MA). MCF7, MCF7-R1, and DMS114 cells were seeded in 96-well plates (5000 cells/well) and cultured in the required media supplemented with 10 U/mL heparin and then treated with various concentrations of FGF2, conjugates, or unconjugated MMAE. After 96 h of continuous exposure, the medium was removed and replaced with a fresh one containing 10% alamarBlue reagent. Then, after 4 h of incubation, fluorescence emission at 590 nm (excitation at 560 nm) was measured using an EnVision Multilabel Reader fluorescence plate reader (PerkinElmer, Waltham, MA). Data are mean values from three independent experiments (each point in a single experiment was evaluated in triplicate) ± SE. The half-maximal effective concentration (EC_50_) was calculated from the concentration–response curve obtained for each preparation.

### In Vivo Anti-Cancer Effects of FGF2-(PEG27-MMAE)_3_ in a Mouse Xenograft Model

The experimental protocol was evaluated and approved by the Norwegian Food Safety Authority (FOTSid 8697) and conducted in compliance with the European Convention of the Protection of Vertebrates Used for Scientific Purposes (EU Directive 2010/63/EU).

#### Animal Information

Female NOD.Cg-*Prkdc^scid^Il2rg^tm1Wjl^*/SzJ (NSG) mice, 3–6 weeks old, were purchased from Jackson Laboratory (cat. number 005557). The mice were housed according to the standard regime at the Department of Comparative Medicine, The Norwegian Radium Hospital, with ad libitum access to food and water and cage changes twice a week. To ensure tumor growth, 4 μg/mL beta-estradiol (Sigma-Aldrich, E8875) was added to the drinking water.

To generate tumors, MCF7-R1 cells (5 × 10^6^) were diluted in 200 μL of serum-free RPMI-1640 media and injected subcutaneously (s.c.) into the right and left flanks of the mice. After approximately 120 days, MCF7-R1 tumors had reached a volume of about 1000 mm^3^. The donor mice were then sacrificed by cervical dislocation, and the MCF7-R1 tumor was extracted and cut into 2 mm^3^ pieces and s.c. implanted bilaterally into the flanks of new NSG mice. Anesthesia was obtained with 4% (v/v) sevofluran (Baxter, Deerfield, IL) along with 1 L/min oxygen and 3 L/min nitrous oxide. After approximately 70 days, the mice were divided into four treatment groups of 4–5 mice, with an average tumor volume of approximately 100 mm^3^ in each group. Each treatment (PBS 10 mL/kg, FGF2 10 mg/kg, MMAE 0.6 mg/kg, and FGF2-(PEG27-MMAE)_3_ 10 mg/kg) was given intravenously (i.v.) in the tail vein once a week for two consecutive weeks. The tumor volume and the body weight were monitored twice a week throughout the experiment. The tumor volume was measured using a caliper, and the volume was calculated as follows: *V* = *W*^2^ × *L* × 0.5 (where *W* and *L* are the shortest and longest tumor diameters, respectively). To generate the tumor growth curves, the tumor volume was normalized to the median tumor volume at the beginning of treatment in each group and plotted over time. Body weights were normalized to the pretreatment weight. Error bars represent the standard error of the mean.

### Spatial Distribution of Conjugation Sites on the Surface of FGF2

The structure of FGF2 with an N-terminal extension of KCKSGG was predicted using the IntFOLD5 server^45^ and visualized by UCSF Chimera 1.15 software.^46^

## Results and Discussion

### Synthesis of PEGylated MMAE Moieties

**Figure 1:**
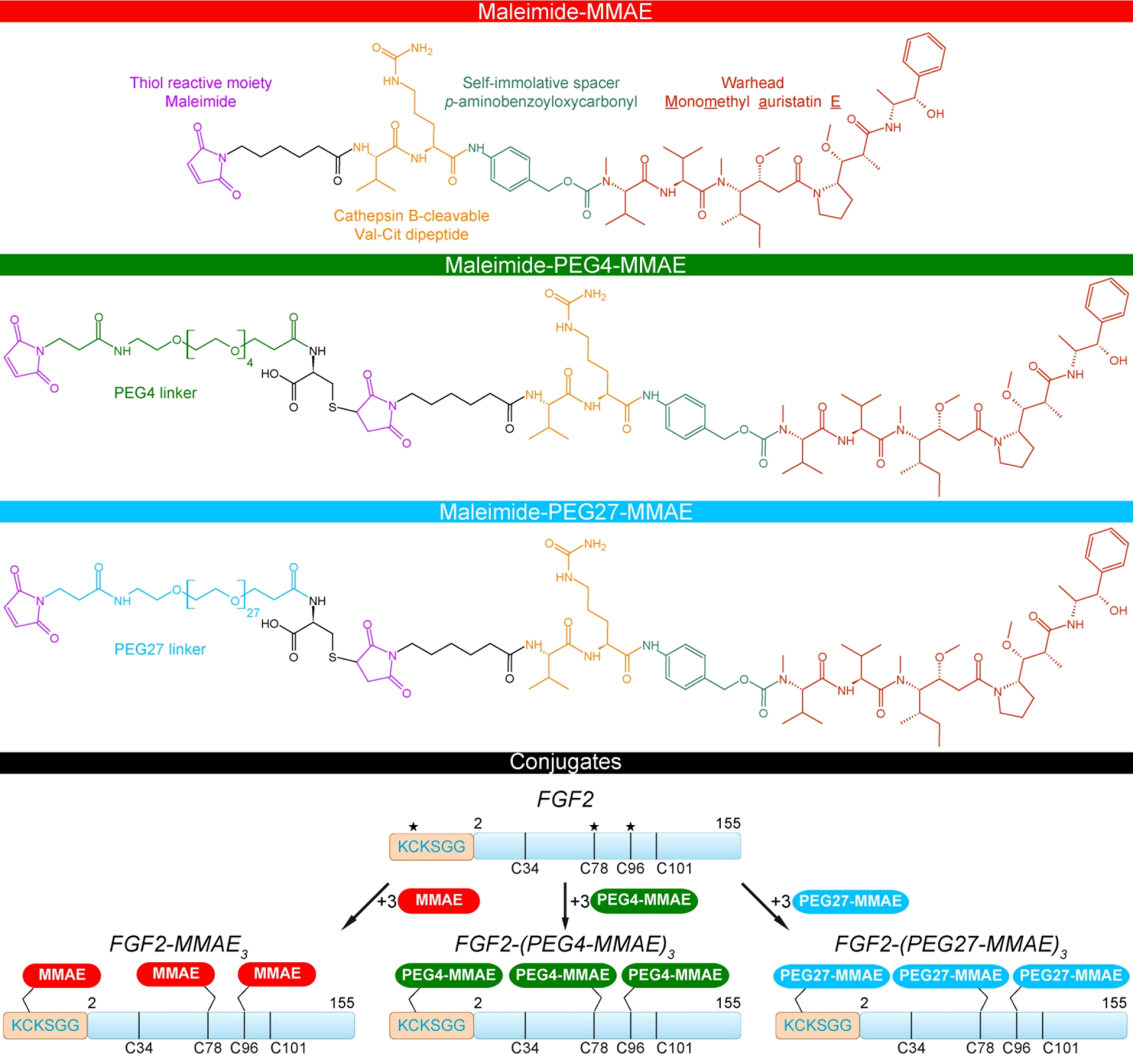
Chemical structures of cytotoxic payloads (MMAE and PEGylated MMAEs) and schematic representation of FGF2 conjugates. The asterisks correspond to the cysteines undergoing conjugation.

Previously, we reported the ability of FGF2 conjugated to a single MMAE molecule to kill several cancer cell lines overproducing fibroblast growth receptor 1.^12,13^ We also showed that a drug-to-protein ratio (DPR) of three provided a greater cytotoxic effect than FGF2 loaded with one or two molecules of MMAE.^12^ Wild-type FGF2 has four cysteine residues, including two buried in the protein core (Cys34 and C101) and two on the surface (Cys78 and Cys96), exposed to the solvent and reacting with, for example, maleimide (Figure 1 and Figure S1). Thus, to obtain a DPR of three, we used a variant of FGF2 with an N-terminal extension of KCKSGG. As we mentioned previously, the lysine-flanked cysteine residue is highly susceptible to the maleimide–thiol reaction.^11,12^

However, the attachment of three highly hydrophobic drug molecules creates serious problems with aggregation of the conjugate. In this study, we used a bifunctional PEG containing an NHS ester and maleimide groups to produce FGF2 conjugated with three MMAE molecules. In addition to reducing hydrophobicity, the use of PEG moieties has two additional advantages: it increases the hydrodynamic radius of relatively small (17.8 kDa) FGF2 molecules and allows conjugation of the cytotoxic payload under mild conditions via two highly reactive groups. In order to investigate the effect of the PEG size on the hydrodynamic properties of the conjugates, we used PEG with either 4 or 27 mers of ethylene oxide. This yielded two conjugates, each containing three MMAE molecules with a cathepsin B-sensitive dipeptide (Val-Cit) linker directly connected to the FGF2 molecule, FGF2-(PEG4-MMAE)_3_, and FGF2-(PEG27-MMAE)_3_ (Figure 1). As a control, we used previously reported FGF2-MMAE_3_.^12^

**Figure 2:**
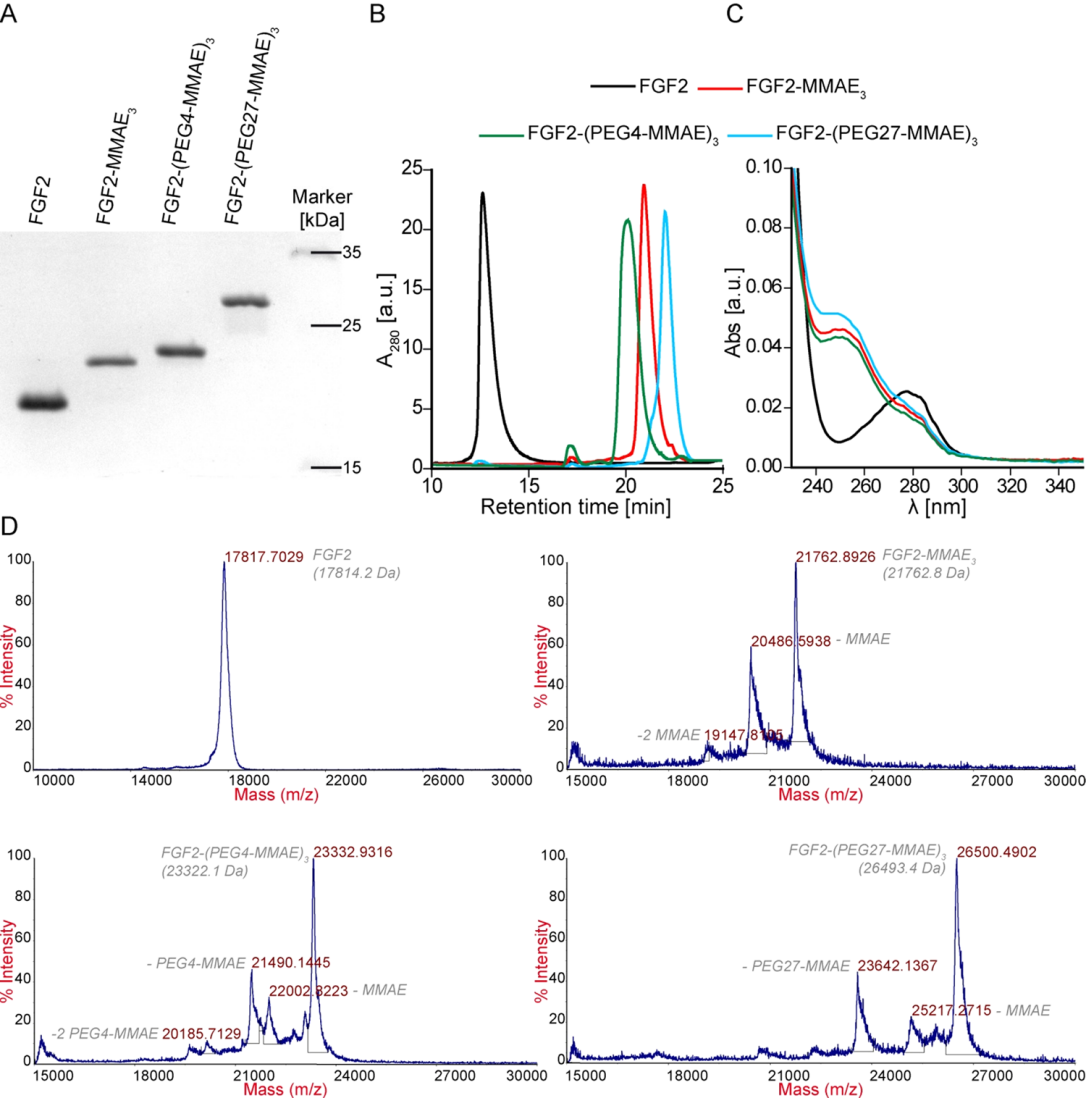
Conjugation of FGF2 to MMAE and PEGylated MMAE. (A) SDS-PAGE analysis of cation exchange-purified conjugation reaction products. (B) Purity and efficiency of conjugation determined by RP-HPLC. (C) DPR quantified by UV–vis. (D) Mass spectra of FGF2 and conjugates recorded by MALDI-MS.

**Figure 3:**
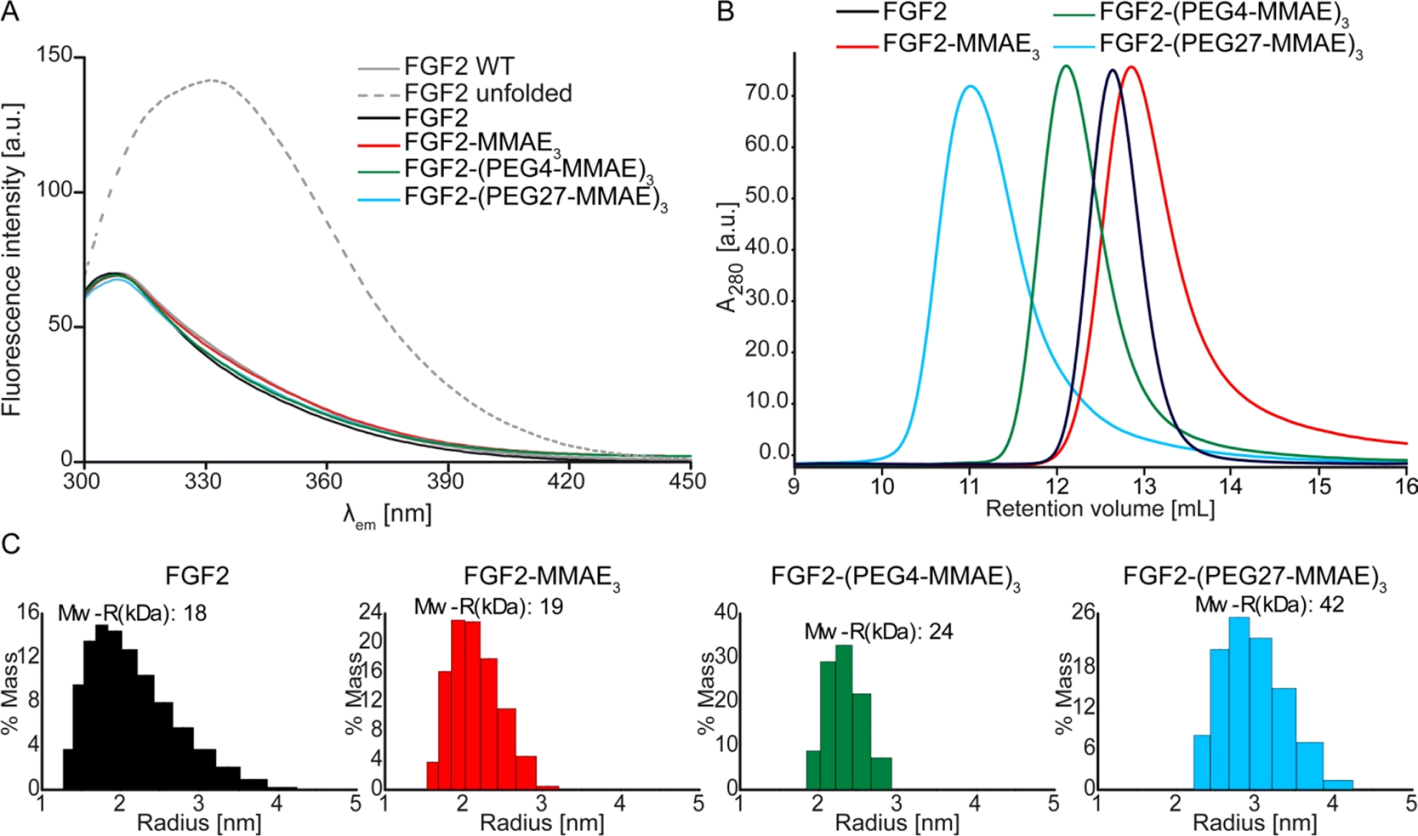
Biophysical analysis of FGF2 conjugates. (A) Fluorescence emission spectra (300–450 nm) of FGF2 conjugates at 4 μM concentration upon excitation at 280 nm. The dashed line represents FGF2 unfolded upon exposure to 80 °C in 3 M GdmCl. (B) Retention volume of FGF2 conjugates determined by SEC. (C) DLS analysis of the protein size distribution. The estimated molecular masses are given in Table 1.

We obtained highly homogenous conjugates that contained almost exclusively triply substituted forms, as shown by SDS-PAGE, RP-HPLC, and UV–vis (Figure 2A–C). The DPR values calculated from the UV–vis measurement are 2.9 for FGF2-MMAE_3_, 3.1 for FGF2-(PEG4-MMAE)_3_, and 3.0 for FGF2-(PEG27-MMAE)_3_ (Figure 3C).^47^ The identity of conjugates was confirmed by MALDI-MS (Figure 2D and Table 1).

**Table 1:** Comparison of FGF2 Molecular Masses Calculated and Determined by MS, SEC, and DLS

	molecular mass			
	calculated	MS	SEC	DLS
preparation	[Da]		[kDa]	
FGF2	17,814.2	17,817.7	17	18
FGF2-MMAE_3_	21,762.8	21,762.9	16	19
FGF2-(PEG4-MMAE)_3_	23,322.1	23,332.9	22	24
FGF2-(PEG27-MMAE)_3_	26,493.4	26,500.5	34	42

### Biophysical and Biological Properties of the Conjugates

To confirm the native state of FGF2 within the conjugates, we performed fluorescence analysis of the protein tertiary structure. Properly folded FGF2 exhibits relatively high emission from tyrosine at around 303 nm and low emission at 353 nm from tryptophan residues, whose emission is quenched by neighboring residues (Figure 3A). After 2 h of FGF2 unfolding in 3 M guanidine hydrochloride at 80 °C, a huge increase in fluorescence emission around 353 nm was observed (Figure 3A). The fluorescence spectra of wild-type FGF2, unconjugated FGF2 with N-terminal extension (KCKSGG), and conjugates were similar. They did not show tryptophan emission, confirming that FGF2 is properly folded after conjugation with MMAE and PEGylated MMAE (Figure 3A).

The effective hydrodynamic mass of each conjugate was determined by SEC using a Superdex G75 column (Figure 3B). Compared with the attachment of three MMAE molecules, the conjugation of three PEG4-MMAE or PEG27-MMAE molecules increased the hydrodynamic mass of the conjugate from 16 to 22 kDa and to 34 kDa, respectively (Figure 3B and Table 1). DLS measurements provided higher values, from 19 to 24 kDa and 42 kDa, respectively (Figure 3C and Table 1). We also found that the average hydrodynamic radius of FGF2-(PEG4-MMAE)_3_ and FGF2-(PEG27-MMAE)_3_ increased to 2.5 and 3 nm in comparison to 2 nm for FGF2-MMAE_3_ (Table 1).

To verify whether attachment of three MMAEs via PEG4 or PEG27 affects the affinity of FGF2 to the extracellular domain of FGFR1 IIIc, we carried out BLI measurements. For all molecules tested, the dissociation constant (*K*_D_) values were in the nanomolar range (Table 2). Direct attachment of MMAE increased the *K*_D_ value (decreased the affinity to receptor) by a factor of 4.2. Attachment of MMAE via PEG4 or PEG27 led to about threefold decrease in affinity.

**Table 2:** Kinetic Constants of Binding of FGF2 and the Conjugates to the Extracellular Domains of FGFR1 IIIc Determined by BLI

preparation	concentration [nM]	*K*_D_ [nM]	*K*_D_® [nM]	*k*_on_ × 10^5^ (1/Ms)	*k*_on_® × 10^5^ [1/Ms]	*k*_dis_ × 10^–4^ [1/s]	*k*_dis_® × 10^–4^ [1/s]	*R*_Max_	*X*^2^
FGF2	40	0.42 ± 0.01	**0.34**	7.29 ± 0.06	**6.33**	3.09 ± 0.06	**2.28**	0.33	0.0352
	60	0.43 ± 0.01		6.56 ± 0.07		2.85 ± 0.07		0.306	0.0414
	80	0.18 ± 0.01		5.15 ± 0.03		0.91 ± 0.04		0.3353	0.0135
FGF2-MMAE_3_	40	1.56 ± 0.02	**1.43**	2.53 ± 0.01	**2.28**	3.94 ± 0.06	**3.24**	0.4331	0.0362
	60	1.00 ± 0.03		2.32 ± 0.01		2.33 ± 0.06		0.3591	0.0299
	80	1.73 ± 0.02		2.00 ± 0.01		3.46 ± 0.03		0.3951	0.0093
FGF2-(PEG4-MMAE)_3_	40	0.65 ± 0.01	**0.90**	3.65 ± 0.01	**2.88**	2.38 ± 0.03	**2.47**	0.5288	0.0236
	60	0.83 ± 0.01		2.68 ± 0.01		2.23 ± 0.04		0.5562	0.0301
	80	1.21 ± 0.02		2.31 ± 0.01		2.80 ± 0.05		0.5744	0.0498
FGF2-(PEG27-MMAE)_3_	40	0.76 ± 0.01	**1.08**	4.18 ± 0.02	**3.34**	3.18 ± 0.05	**3.41**	0.575	0.0489
	60	0.97 ± 0.02		3.29 ± 0.02		3.20 ± 0.06		0.5415	0.0697
	80	1.52 ± 0.02		2.54 ± 0.01		3.85 ± 0.05		0.5548	0.0555

**Figure 4:**
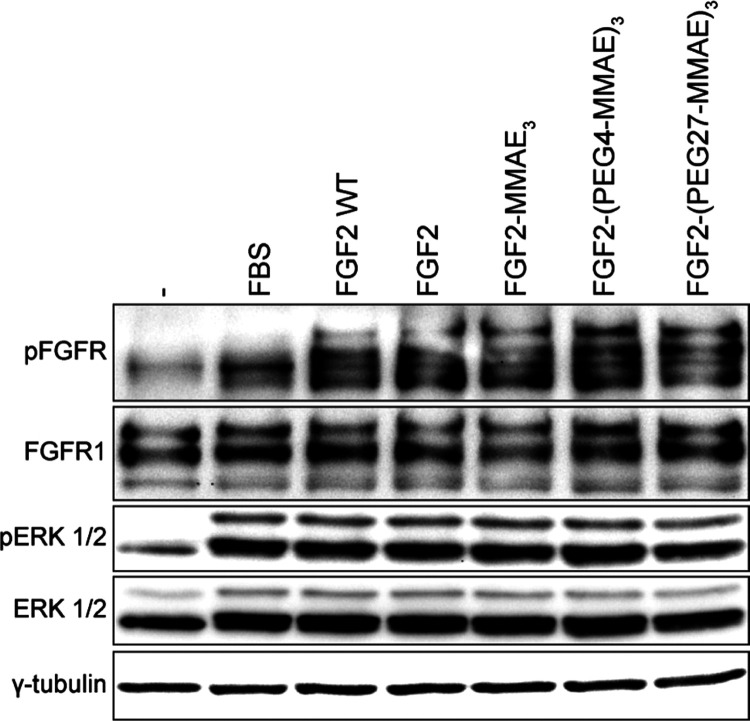
Activation of downstream signaling in NIH 3T3 cells after 15 min of stimulation with 15 ng/mL FGF2 variants or their conjugates in the presence of 10 U/mL heparin detected by Western blotting using anti-phospho-FGFR (pFGFR) and anti-phospho-ERK1/2 (p-ERK1/2) antibodies. The total amount of FGFR1, ERK 1/2, and γ-tubulin served as loading controls.

We also studied whether FGF2 conjugates can activate FGFR-dependent signaling pathways in NIH 3T3 cells. Serum-deprived cells were incubated for 15 min with 15 ng/mL FGF2 proteins and FGF2 conjugates. Western blotting analysis of phospho-FGFR and phospho-ERK 1/2 shows that FGF2 conjugates activated downstream signaling at the same level as the wild type of FGF2 (Figure 4), indicating that MMAE conjugated either directly or via PEG4/27 had no effect on the interaction of FGF2 with its cellular target. To further investigate whether conjugation does not affect binding of FGF2 to FGFR1, we examined the activation of downstream signaling in NIH 3T3 cells at different concentrations of FGF2 conjugates. As shown in Figure S2, for the concentrations tested (0.1–15 ng/mL), there were no differences in FGFR1 and ERK1/2 phosphorylation between FGF2 WT and FGF2-based conjugates.

### Internalization of FGF2 Conjugates

**Figure 5:**
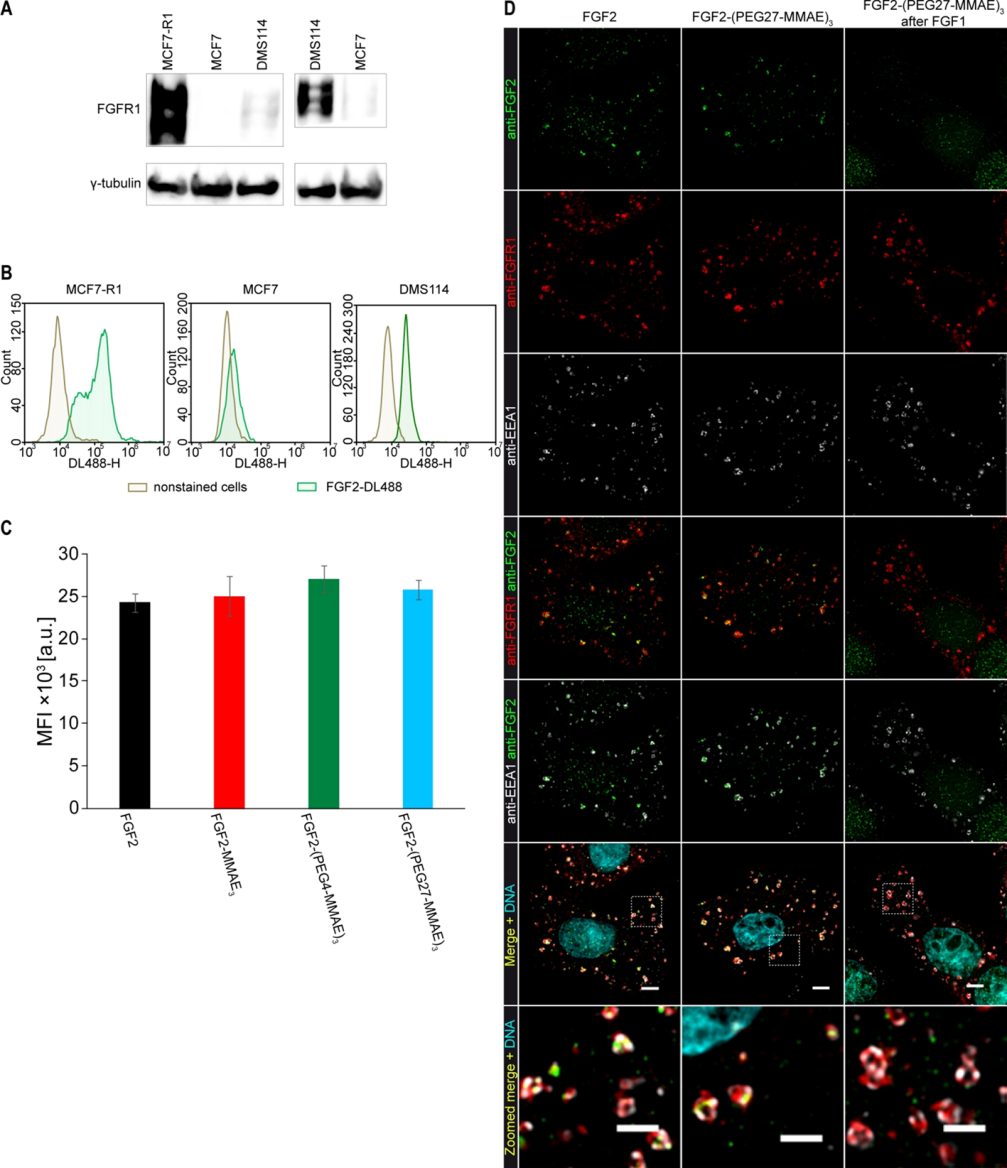
FGFR1 levels and internalization efficiency of FGF2 and its conjugates in MCF7-R1, MCF7, and DMS114 cells. (A) Expression level of FGFR1 in MCF7-R1, MCF7, and DMS114 cell lines. Equal numbers of cells were separated by SDS-PAGE and subjected to Western blotting analysis. (B) Flow cytometry analysis of internalization efficiency in MCF7-R1, MCF7, and DMS114 cell lines. Cells were incubated on ice with 500 ng/mL FGF2 labeled with DyLight488 for 60 min. Then cells were moved to 37 °C for 40 min and subsequently analyzed by flow cytometry. (C) Quantitative analysis of internalization of FGF2 and its conjugates in MCF7-R1 cells. Cells were incubated with FGF2 or its conjugates labeled with DyLight488 and analyzed by flow cytometry. Data shown are mean fluorescence intensities (MFI) from three independent experiments ±SD. A one-way ANOVA test was performed, and the differences between groups were statistically insignificant. (D) Fluorescence microscopy analysis of FGF2 and FGF2-(PEG27-MMAE)_3_ endocytosis in MCF7-R1 cells. FGF2 (left column) or FGF2-PEG27-MMAE (middle column) were incubated with cells at 4 °C for 60 min and then at 37 °C for 40 min to allow internalization. Cells shown in the right column were preincubated with FGF1 at 4 °C for 10 min and then incubated with FGF2-(PEG27-MMAE)_3_ at 4 °C for 60 min before incubation at 37 °C for 40 min. Cells were then fixed and stained with anti-FGF2 (green), anti-FGFR1 (red), anti-EEA1 (white) antibodies, and Hoechst 33342 to visualize DNA. The squares marked in the four-color overlay images indicate blown-up regions enlarged and shown in the bottom row. The bar corresponds to 4 μm and to 2 μm in zoomed images.

The cytotoxic effect of a protein–drug conjugate largely depends on its efficient receptor-dependent internalization into the cell. As a first step, we checked FGFR1 levels in MCF7 cells stably transfected with FGFR1 (MCF7-R1), MCF7, and DMS114 cells by Western blotting analysis of the whole cell lysate. As shown in Figure 5A, MCF7-R1 cells show a significantly augmented FGFR1 level compared with DMS114 cells having a naturally high level of FGFR1 expression. In contrast, the MCF7 cell line produces trace amounts of FGFR1 (Figure 5A).

Then we analyzed the efficiency of internalization of fluorescently labeled FGF2 into MCF7-R1, MCF7, and DMS114 cells by flow cytometry. As shown in Figure 5B, the highest signal of fluorescently labeled FGF2 was detected in MCF-R1 cells. A significantly lower signal was observed in DMS114 cells. Untransfected MCF7 cells displayed weak fluorescent signals of labeled FGF2. These data correlate with the FGFR1 levels detected by Western blotting (Figure 5A) and indicate a strong correlation between the level of receptor and internalization efficiency.

In the next step, we tested whether the conjugation with MMAE and PEGylated MMAE affects the efficiency of FGF2 endocytosis. Figure 5C shows the flow cytometry quantification of the internalization of FGF2 and its conjugates into MCF7-R1 cells. We observed that MMAE or PEGylated MMAE did not influence FGF2 internalization.

Finally, using fluorescence microscopy, we verified that the FGF2-(PEG27-MMAE)_3_ conjugate was internalized as efficiently as FGF2 in MCF7 cells stably transfected with FGFR1 (MCF7-R1). To this end, MCF7-R1 cells were incubated with FGF2 or FGF2-(PEG27-MMAE)_3_ at 4 °C and next kept at 37 °C for 40 min. Then the cells were fixed and stained by an antibody against FGF2, FGFR1, and early-endosome antigen 1 (EEA1). Figure 5D shows that both FGF2 and the FGF2-(PEG27-MMAE)_3_ conjugate were localized into intracellular vesicles and most of these vesicles were positive for EEA1, a membrane-bound marker of early endosomes. Both FGF2 and the conjugate colocalized with FGFR1. These results indicate that FGF2-(PEG27-MMAE)_3_ and FGF2 are efficiently and specifically endocytosed by an FGFR1-mediated mechanism.

We also tested whether receptor saturation by FGF1 can block internalization of the conjugate. First, MCF7-R1 cells were preincubated at 4 °C with a 10-fold excess of FGF1 and then FGF2-(PEG27-MMAE)_3_ was added. Cells were then transferred to 37 °C for 40 min, fixed, and stained as described above. We did not observe the presence of the FGF2 conjugate inside the cells (Figure 5D). This experiment supports the conclusion that FGF2-(PEG27-MMAE)_3_ is effectively endocytosed in an FGFR1-dependent manner in MCF7-R1 cells as uptake can be efficiently competed out by the FGFR1 ligand, FGF1.

### In Vitro Cytotoxicity of FGF2 Conjugates

To evaluate in vitro the inhibitory effects of FGF2 conjugates on cell growth, we used FGFR1-positive cell lines: DMS 114 (small cell lung cancer, SCLC)^15^ and MCF7 (human breast adenocarcinoma) cells stably transfected with FGFR1 (MCF7-R1). MCF7 cells, expressing a relatively low level of FGFR1, served as a control cell line.

**Figure 6:**
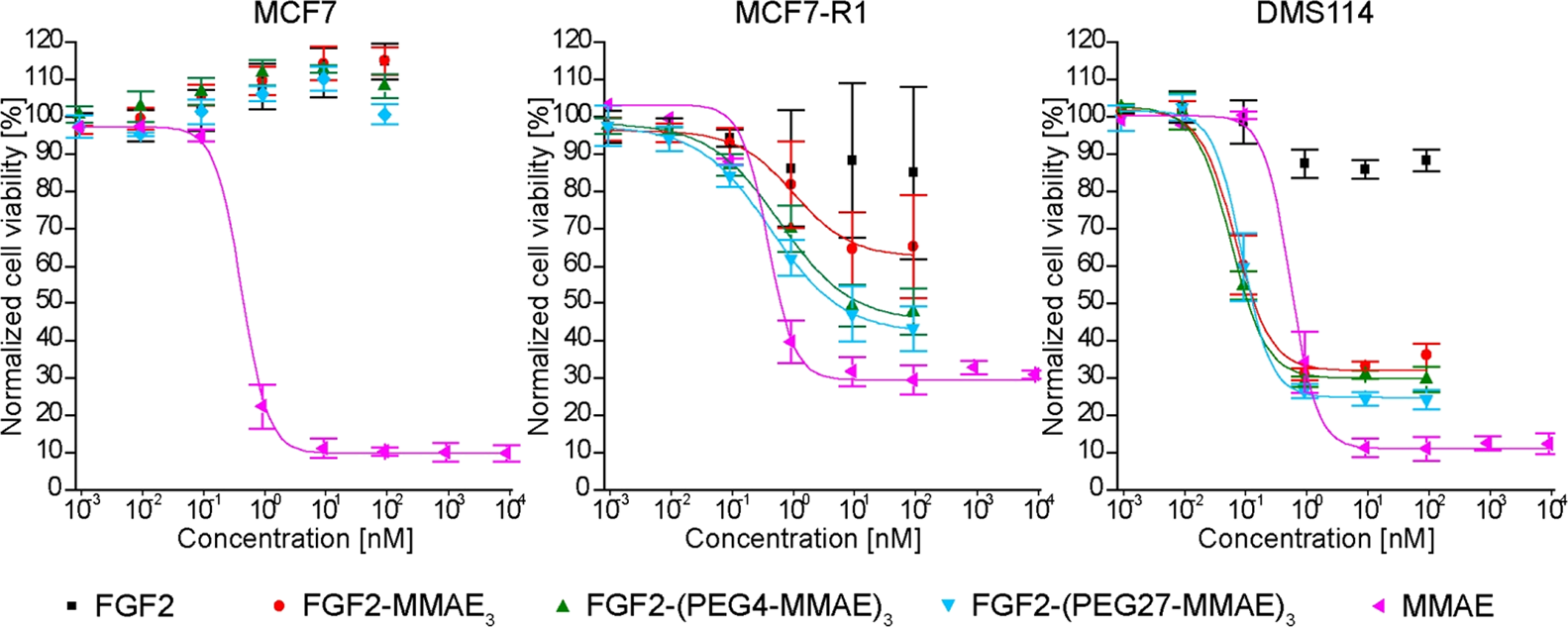
Viability of cells treated with FGF2, FGF2 conjugates, and free MMAE. MCF7-R1, DMS114 (FGFR1-positive), and MCF7 (with low FGFR1 level) cell lines were treated with the indicated agents for 96 h, and then the cell viability was assessed using the alamarBlue reagent. Data shown are mean values from three experiments ±SD. Solid lines represent Hill’s equation fits.

For FGFR1-positive cells, the conjugates showed a strong cytotoxic effect. In the case of MCF7-R1 cells, both PEGylated conjugates (via PEG4 and PEG27) were about twofold more toxic than the non-PEGylated FGF2-MMAE_3._ Their cytotoxicity was comparable to that of free MMAE (Figure 6 and Table 3). However, for DMS114 cells, all three conjugates (two PEGylated and FGF2-MMAE_3_) showed similar EC_50_ values, in the low nanomolar range, which were about eightfold lower than those of free MMAE (Figure 6 and Table 3). No toxic effects of all three conjugates were observed in MCF7 cells. Free MMAE exhibited a high cytotoxic effect at the subnanomolar level (EC_50_ = 0.41 nM) (Figure 6 and Table 3). These results demonstrate high specificity and the potency of FGF2 conjugates in killing FGFR1-expressing cells.

**Table 3:** Cytotoxicity of FGF2 Conjugates and Free MMAE in Different Cell Lines^a^

	preparation			
	FGF2-MMAE_3_	FGF2-(PEG4-MMAE)_3_	FGF2-(PEG27-MMAE)_3_	MMAE
cell line	EC_50_ [nM]			
MCF7	>91★★★	>91★★★	>91★★★	0.41 ± 0.02
MCF7-R1	1.01 ± 0.42★	0.59 ± 0.19★	0.42 ± 0.03★	0.38 ± 0.05
DMS114	0.09 ± 0.03★★	0.07 ± 0.01★★	0.06 ± 0.02★★	0.54 ± 0.07

a Student’s t-test was used to determine whether the differences between FGF2-conjugates and free MMAE are statistically significant (*p* values: ∗, 0.05 > *p* ≥ 0.01; ∗∗, 0.01 > *p* ≥ 0.001; ∗∗∗, *p* < 0.001), *n* = 3.

Furthermore, we examined the mechanism of cell killing by MMAE and conjugates using the Annexin V–PI assay (Figure S3). We observed that after 72 h of treatment of MCF7-R1 cells with 10 nM conjugates, the cells were mainly in the early stage of apoptosis. In the case of free MMAE, the percentage of early apoptotic cells was lower, in favor of late apoptotic/necrotic cells. In all treatment groups, the level of viable cells was at a comparable level (approximately 20%).

### FGF2-(PEG27-MMAE)_3_ Inhibits Tumor Growth In Vivo

**Figure 7:**
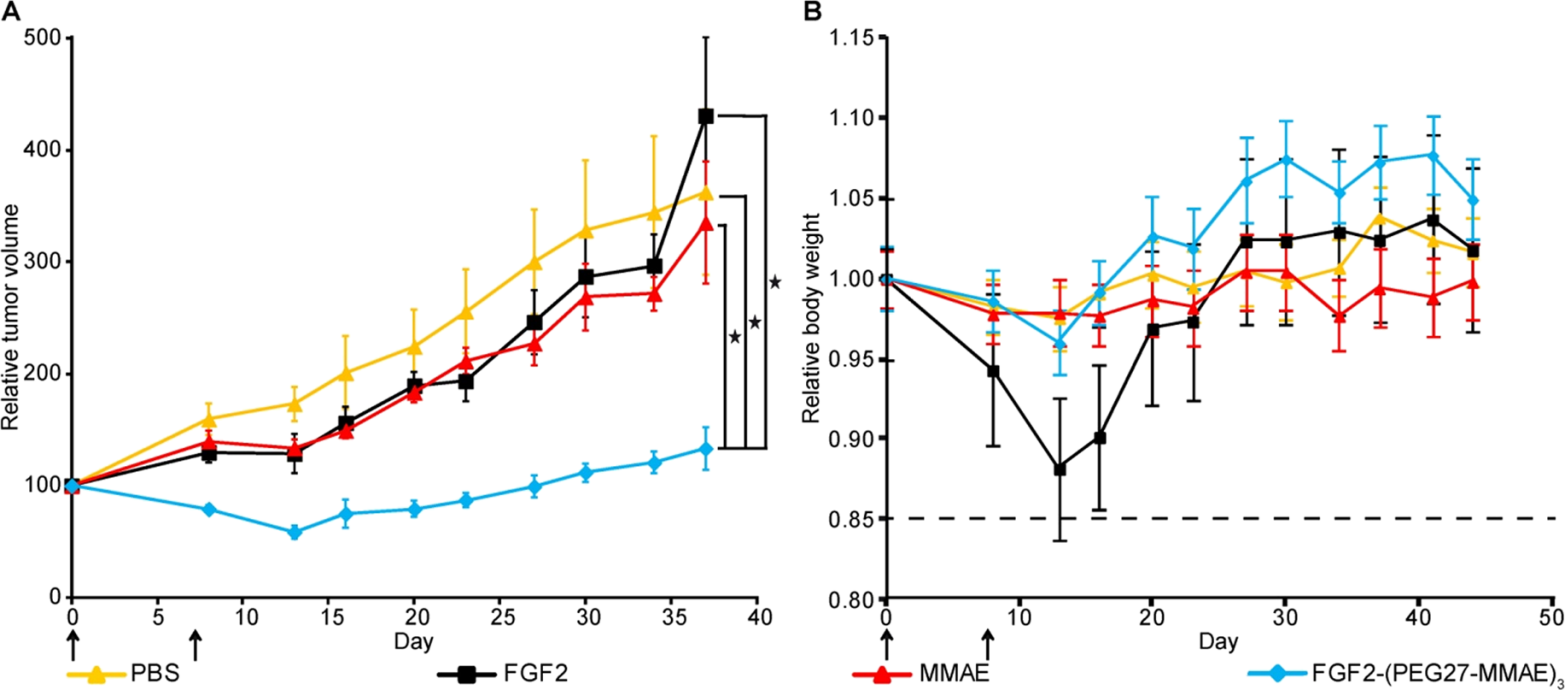
Treatment efficacy and toxicity in mice bearing MCF7-R1 tumors. NSG mice (four mice per group) were treated once a week for two consecutive weeks (day 0 and 7) with intravenous injections of 10 mL/kg PBS, 10 mg/kg FGF2, 0.6 mg/kg MMAE, and 10 mg/kg FGF2-(PEG27-MMAE)_3_, (A) growth inhibitory effect measured as relative tumor volume. (B) Relative body weight of the treated mice. Error bars represent the standard error of the mean. The arrows indicate the day of administration of the preparations, and the dashed line shows the safe level of weight loss. A Student’s t-test was used to determine the statistical significance (*p* values: ∗, 0.05 > *p* ≥ 0.01).

To investigate the anti-tumor effect of FGF2-(PEG27-MMAE)_3_ in vivo, MCF7-R1 cells overproducing FGFR1 were injected into the flanks of NSG mice to generate a human tumor. When tumors reached a size of approximately 100 mm^3^, the mice were randomized into the following treatment groups: vehicle (PBS), empty carrier (FGF2), free MMAE, or FGF2-(PEG27-MMAE)_3_ conjugate. The conjugate was administered once a week for two consecutive weeks at a concentration of 10 mg/kg body weight, corresponding to a drug dose of 0.6 mg/kg body weight of free MMAE. Free auristatin or FGF2 had no effect on the tumor, whereas FGF2-(PEG27-MMAE)_3_ very strongly inhibited the tumor growth, as shown in Figure 7A. Furthermore, it was observed that a higher dose of free MMAE (1.2 mg/kg) caused a dangerous decrease in the body weight (Figure S4). Administration of FGF2-(PEG27-MMAE)_3_ did not cause any body weight loss or other side effects in the animals during the treatment period. However, a reversible weight loss of about 10% was observed in the mice treated with FGF2 (Figure 7B).

We previously described conjugates of FGF2 with one, two, or three molecules of hydrophobic vcMMAE, which exhibited a highly cytotoxic effect against FGFR1-overproducing cell lines, depending on the number of cytotoxic payloads per FGF2 molecule. Triply loaded FGF2-vcMMAE showed the highest cytotoxicity, but due to its increased hydrophobicity, it tended to precipitate and had to be stored at concentrations below 1.5 mg/mL.^12^

In this study, we developed a more hydrophilic version of FGF2-MMAE_3_ by introducing a spacer (PEG4 or PEG27) between FGF2 and vcMMAE, which increased the hydrodynamic radius of PEG-containing conjugates and favorably affected the pharmacokinetic properties of the conjugates. In comparison with non-PEGylated FGF2-MMAE_3_, the introduction of a small PEG4 linker increased the apparent molecular mass from 16 to 22 kDa, as determined by SEC (Figure 3 and Table 1). A much larger increase was found for the PEG27 spacer (from 16 to 34 kDa) (Figures 2 and 3 and Table 1). It should be stressed that a similarly large increase in molecular mass was observed for triply substituted FGF2-PEG2-MMAE in the DLS analysis. Again, the increase in the apparent molecular mass for FGF2-(PEG4-MMAE)_3_ was much less pronounced. Both SEC and DLS data show that the size of FGF2-(PEG27-MMAE)_3_ is sufficient to overcome renal clearance.^48,49^ The radius of the FGF2-(PEG27-MMAE)_3_ conjugate determined by DLS is approximately 3 nm, which ensures good tumor penetration, in contrast to a full-length antibody or nanoparticles with radii of 10 and 100 nm, respectively.^50,51^ Our observations are consistent with recent studies of Seattle Genetics company, which showed that the PEG4 linker is insufficient, whereas PEG chains longer than 8 units improve pharmacokinetics and reduce renal clearance of ADCs.^52^ Similarly, Lyon et al. showed that reduced hydrophobicity improves pharmacokinetics and expands the therapeutic windows of ADCs highly loaded with MMAE.^53^ Furthermore, Simmons et al. showed that the improvement in PK, tolerability, specificity, and expansion of the therapeutic window of PEGylated conjugates is mainly due to a reduction in nonspecific interactions.^54^ Our DLS analysis shows that the non-PEGylated conjugate has a slightly greater hydrodynamic radius than FGF2 (1.8 versus 2.2 nm) but migrates more slowly in the SEC (Figure 3 and Table 1), suggesting that FGF2-MMAE_3_, but not its PEGylated variants, makes nonspecific hydrophobic interactions.

It has been reported that, in several cases, PEG molecules affect specific interactions of biomolecules with molecular targets.^55,56^ In agreement, Zhao et al. observed that PEGylation of FGF2 at sites located in close proximity to the receptor-binding region decreased the affinity to the receptor and heparin and affected its biological activities, such as activation of downstream signal transduction and stimulation of proliferation.^57^ We conjugated PEGylated drugs to cysteines separated from the primary and secondary FGFR1 binding sites^58^ (Figure S1). We validated that PEG4- and PEG27-based conjugates did not affect the interaction with isolated extracellular domains of FGFR1 (Table 2) as well as FGFR1 on the surface of NIH 3T3 cells (Figure 4 and Figure S2).

Proper interaction of the conjugates with FGF receptors ensures efficient internalization and FGFR-dependent trafficking to endosomes and then lysosomes, which is crucial for MMAE release and cell-killing effect^32,59,60^ As shown in Figure 5, all conjugates were internalized at the same level as FGF2. Both FGF2 and FGF2-(PEG27-MMAE)_3_ were internalized into the cell interior and colocalized with FGFR1 in endosomes. Furthermore, after cell surface saturation with FGF1, no internalization of FGF2-PEG27-MMAE_3_ was observed (Figure 5D right column), further ruling out an FGFR1-independent pathway of conjugate endocytosis. This is very important since MMAE equipped with a Val-Cit dipeptide and a PAB spacer requires lysosome-associated cathepsin B to release the active form of cytotoxin.^61^

Both FGF2-(PEG4-MMAE)_3_ and FGF2-(PEG27-MMAE)_3_ exhibited high cytotoxicity against FGFR1-positive cell lines (DMS114 and MCF7-R1) in in vitro assays (EC_50_ values from subnanomolar to nanomolar range) and were nontoxic to the FGFR1-negative cell line (MCF7) (Figure 6 and Table 3). The DMS114 cell line showed the highest sensibility to the conjugates, with no significant differences between PEG4- and PEG27-based conjugates. These conjugates were at least six times more toxic than free MMAE (Figure 6 and Table 3). In the case of the MCF7-R1 cell line, introduction of the PEG27 linker resulted in approximately twofold higher cytotoxicity (FGF2-MMAE_3_ versus FGF2-(PEG27-MMAE)_3_) (Figure 6 and Table 3). Furthermore, we showed that FGF2-based conjugates induce death mainly via apoptosis rather than necrosis or other mechanisms (Figure S3).

Finally, we show that administration of the FGF2-(PEG27-MMAE)_3_ conjugate at a dose of 10 mg/kg on day 0 and a second equal dose on day 7 resulted in a noticeable reduction in the tumor volume. On day 13, the relative tumor volume in the conjugate-treated group had decreased to 58% of the initial size. Further reduction in the tumor volume was not observed, but tumor growth was stunted. Administration of free MMAE in contrast to the FGF2-PEG27-MMAE_3_ conjugate did not inhibit tumor growth. Moreover, FGF2-PEG27-MMAE_3_ did not significantly affect the body weight of mice (Figure 7B), which supports the efficacy of PEGylated FGF2 conjugates as an anti-cancer agent targeting tumors with FGFR1 overproduction.

## Conclusions

We show for the first time the effect of an FGF2-based conjugate on the growth of the human breast cancer cell line MCF7-R1, overexpressing FGFR1, which is grown as a xenograft in NSG mice. So far, only one ADC (Aprutumab Ixadotin, BAY 1187982) targeting the FGF receptor (FGFR2) has been tested in phase I clinical trials, but it was poorly tolerated by patients and is, consequently, no longer under consideration.^62^ The promising data presented in this study show that our FGF2-based PEGylated conjugate should be further developed for targeting FGFR1-positive tumors.

## Supporting Information Available

Spatial distribution of conjugation sites on the surface of FGF2, analysis of concentration-dependent activation of downstream signaling in NIH 3T3 cells, mechanism of killing by MMAE and FGF2-based conjugates verified by Annexin V and the propidium iodide assay, and determination of the maximum tolerated dose of free MMAE in NSG mice (PDF)

## Abbreviations

ADC: antibody–drug conjugate
BLI: biolayer interferometry
DLS: dynamic light scattering
DPR: drug-to-protein ratio
FGF: fibroblast growth factor
FGFR: fibroblast growth factor receptor
MFI: mean fluorescence intensities
MMAE: monomethyl auristatin E
PEG: poly(ethylene oxide)
