# Supplementary material for: Fibroblast Growth Factor 2 Conjugated with Monomethyl Auristatin E Inhibits Tumor Growth in a Mouse Model
Source: Biomacromolecules. 2021 Sep 20;22(10):4169–80. doi: 10.1021/acs.biomac.1c00662 (PMC8512659; doi:10.1021/acs.biomac.1c00662)
Supplement: Supplementary file 1 — bm1c00662_si_001.pdf [file bm1c00662_si_001.pdf]

Supporting Information

Fibroblast Growth Factor 2 Conjugated with Monomethyl Auristatin E Inhibits Tumor Growth in a Mouse Model

Mateusz A. Krzyscik,[†] Malgorzata Zakrzewska,[†] Vigdis Sørensen,[‡,§] Geir Frode Øy,[‖] Skjalg Brunheim,[‖] Ellen M. Haugsten,[‖,§] Gunhild M. Mælandsmo,[‖,¥] Antoni Wiedlocha,[Δ,§,∂] and Jacek Otlewski*[,†]

[†] Department of Protein Engineering, Faculty of Biotechnology, University of Wroclaw, Joliot-Curie 14a, 50-383 Wroclaw, Poland

[‡] Advanced Light Microscopy Core Facility, Dept. Core Facilities, Institute for Cancer Research, The Norwegian Radium Hospital, Oslo University Hospital, Montebello, 0379 Oslo, Norway

[§] Centre for Cancer Cell Reprogramming, Institute of Clinical Medicine, Faculty of Medicine, University of Oslo, Montebello, 0379 Oslo, Norway

[‖] Department of Tumor Biology, Institute for Cancer Research, The Norwegian Radium Hospital, Oslo University Hospital, Montebello, 0379 Oslo, Norway

[Δ] Department of Molecular Cell Biology, Institute for Cancer Research, The Norwegian Radium Hospital, Oslo University Hospital, Montebello, 0379 Oslo, Norway

[¥] University in Tromso - Arctic University of Norway, Tromso, Norway

[∂] Military Institute of Hygiene and Epidemiology, 01-163 Warsaw, Poland

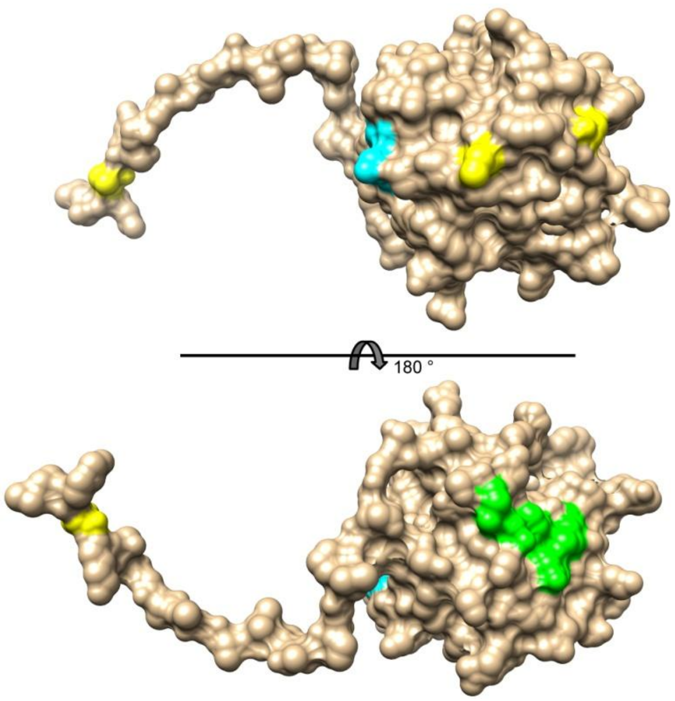

Figure S1. Spatial distribution of conjugation sites on the surface of FGF2. The structure of FGF2 with N-terminal extension of KCKSGG was predicted using the IntFOLD5 server and visualized by UCSF Chimera 1.15 software. Conjugation sites (surface-exposed Cys residues) are shown in yellow, primary FGFR1 binding sites in green, and secondary FGFR1 binding sites in cyan.

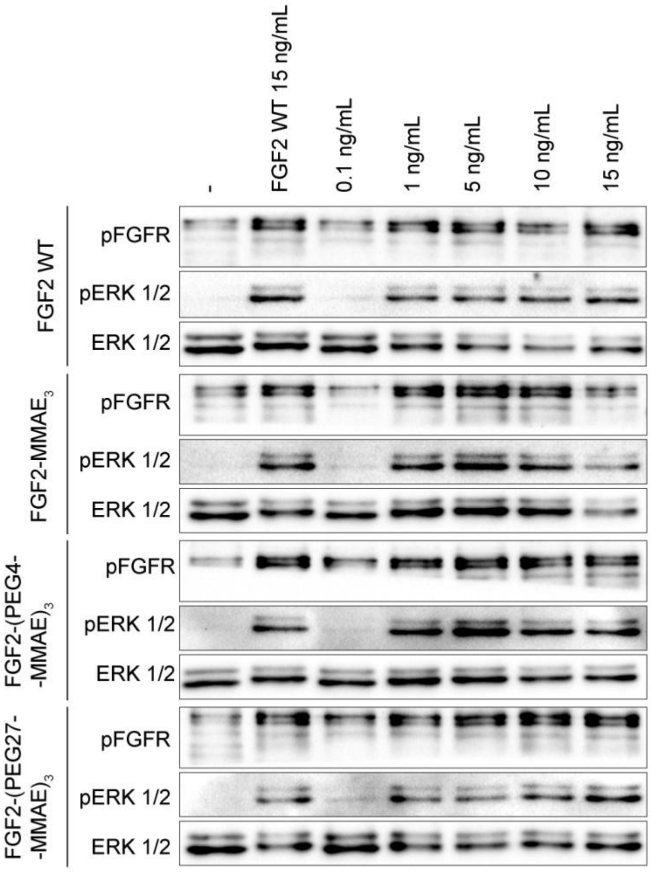

Figure S2. Analysis of concentration-dependent activation of downstream signaling in NIH 3T3 cells after 15 min stimulation with 0.1, 1, 5, 10, 15 ng/mL FGF2 WT or FGF2-based conjugates in the presence of 10 U/mL heparin detected by western blotting using anti-phospho-FGFR (pFGFR) and anti-phospho-ERK1/2 (p-ERK1/2) antibodies. Total ERK 1/2 served as a loading control.

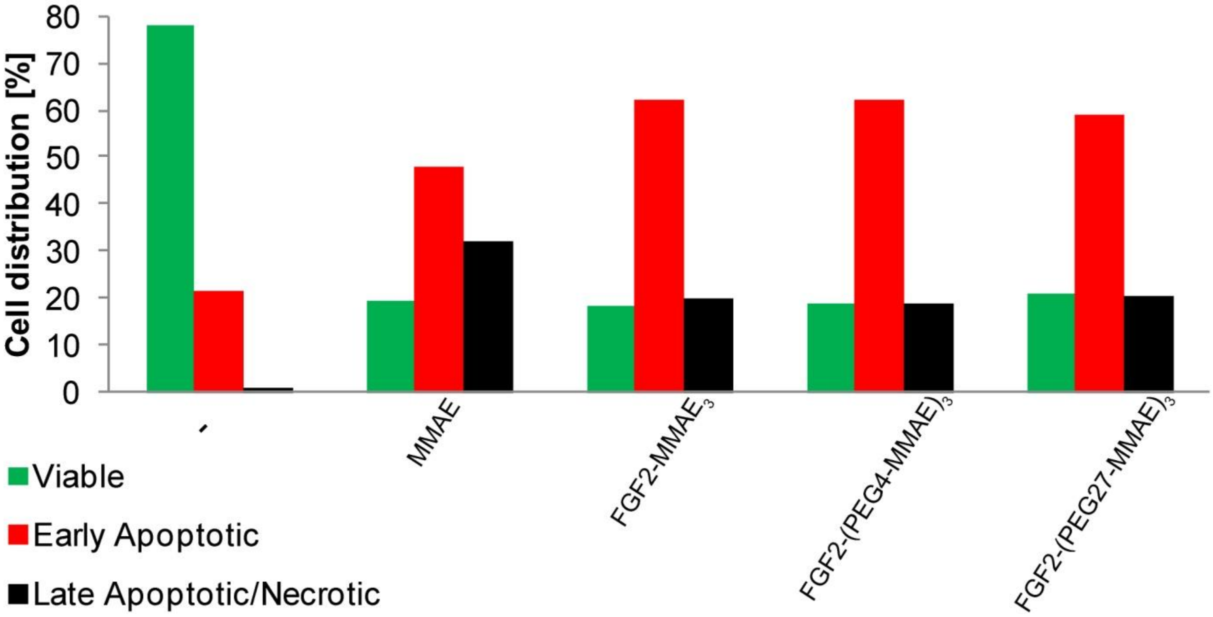

Figure S3. The mechanism of killing by MMAE and FGF2-based conjugates was verified by Annexin V and propidium iodide assay. MCF7-R1 cells were treated with 10 nM of MMAE, $FGF2\text{-}MMAE_3$, FGF2-(PEG4-MMAE)$_3$, or FGF2-(PEG27-MMAE)$_3$ for 72 h, then cells were stained with Annexin V-FITC and propidium iodide and then analyzed by flow cytometry.

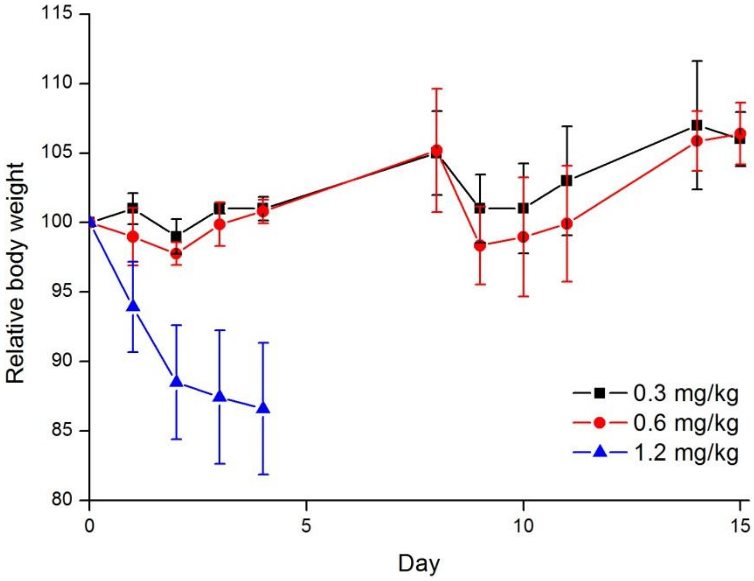

Figure S4. Determination of the maximum tolerated dose of free MMAE in NGC mice. Free MMAE was administered (i.v.) as a single dose (day 0) to three mice per group at a concentration of 0.3, 0.6 or 1.2 mg/kg body weight. Then body mass was monitored. A decrease in body weight greater than 15% resulted in termination of the experiment.
